# Update on Transplacental Transfer of IgG Subclasses: Impact of Maternal and Fetal Factors

**DOI:** 10.3389/fimmu.2020.01920

**Published:** 2020-09-11

**Authors:** Toby Clements, Thomas F. Rice, George Vamvakas, Sara Barnett, Megan Barnes, Beverly Donaldson, Christine E. Jones, Beate Kampmann, Beth Holder

**Affiliations:** ^1^Department of Metabolism, Digestion and Reproduction, Institute of Reproductive and Developmental Biology, Imperial College, London, United Kingdom; ^2^Section of Paediatrics, Division of Infectious Diseases, Department of Medicine, Imperial College, London, United Kingdom; ^3^Department of Biostatistics and Health Informatics, Institute of Psychiatry, Psychology and Neuroscience, Kings College, London, United Kingdom; ^4^Faculty of Medicine and Institute for Life Sciences, University of Southampton and University Hospital Southampton NHS Foundation Trust, Southampton, United Kingdom; ^5^The Vaccine Center, London School of Hygiene and Tropical Medicine, London, United Kingdom; ^6^Vaccines and Immunity Theme, MRC Unit the Gambia at LSHTM, Banjul, Gambia

**Keywords:** pregnancy, placenta, maternal vaccination, antibody, IgG, immunology, infection, neonatal

## Abstract

Transplacental antibody transfer from mother to fetus provides protection from infection in the first weeks of life, and the four different subclasses of IgG (IgG1, IgG2, IgG3, and IgG4) have diverse roles in protection against infection. In this study, we evaluated concentrations and transplacental transfer ratios of the IgG subclasses in a healthy UK-based cohort of mother-cord pairs, and investigated associations with maternal, obstetric, and fetal factors. In agreement with previous studies, we found a strong association between maternal and cord IgG for all subclasses. We report a transfer efficiency hierarchy of IgG1>IgG3>IgG4=IgG2 in our study population, and our review of the literature demonstrates that there is no consensus in the hierarchy of subclass transfer, despite the commonly made statement that the order is IgG1>IgG4>IgG3>IgG2. We report additional data regarding negative associations between elevated maternal IgG concentrations and maternal/cord transfer ratios, finding an effect on IgG1, IgG2, and IgG3 subclasses. Levels of IgG subclasses were the same between venous and arterial blood samples from the umbilical cord, but there was a significantly higher level of total IgG in arterial blood. We found no correlation between placental FcRn protein levels and IgG transfer in our cohort, suggesting that IgG is the main determinant of observed differences in transplacental transfer ratios at term. Neonatal IgG1 and IgG4 levels were increased with later gestation at delivery, independent of any increase in transplacental transfer, indicating that the benefit of later gestation is through accumulation of these subclasses in the fetus. Neonatal IgG2 levels and transfer ratios were reduced in rhesus-negative pregnancies, suggesting that administered anti-D antibodies may compete for transplacental transfer of this subclass. Maternal influenza vaccination resulted in elevated maternal and neonatal levels of IgG4, whereas maternal Tdap vaccination had no impact on neonatal levels of the subclasses, nor transfer. However, within Tdap vaccinated pregnancies, later gestation at Tdap vaccination was associated with higher transplacental transfer. Our study provides information regarding levels and transfer of IgG subclasses in healthy term pregnancies and demonstrates the importance of recording detailed clinical information in studies of antibody transfer, including parity, ethnicity, and timing of maternal vaccine delivery.

## Introduction

The neonatal period is a high-risk period for infectious diseases. Neonates are afforded some protection through passive immunity provided by maternal immunoglobulin (IgG) actively transferred across the placenta during pregnancy. This phenomenon has enabled maternal vaccination strategies aimed at boosting fetal levels of antigen-specific IgG for diseases associated with high neonatal mortality and morbidity. Maternal administration of tetanus and pertussis vaccines have both been highly successful at reducing rates of neonatal disease (1–4).

Transfer of IgG across the placental barrier is thought to involve interaction between IgG and the neonatal Fc receptor (FcRn) in the placental syncytiotrophoblast (5). FcRn is a heterodimer, consisting of an integral membrane glycoprotein which is structurally related to an MHC class I molecule and β2-microglobulin, and is capable of binding the IgG Fc region (6). The binding of FcRn to IgG only occurs at acidic pH 6 (7). For FcRn to bind IgG, IgG must therefore be taken up from the maternal circulation by the syncytiotrophoblast into endosomes which undergo acidification. The endosomes then fuse with the fetal side of the syncytiotrophoblast, leading to an increase in the pH, and the release of IgG into the stroma (8). It is not currently known how IgG crosses the stroma and the second placental barrier—the fetal endothelium—but FcRn may also be present in this endothelial layer (9). Other receptors or Fc-binding proteins found in the placenta could also be involved (6). More detail on the process of IgG transfer is available in our recently published review on FcRn-mediated transplacental antibody transfer (10).

**Figure 1:**
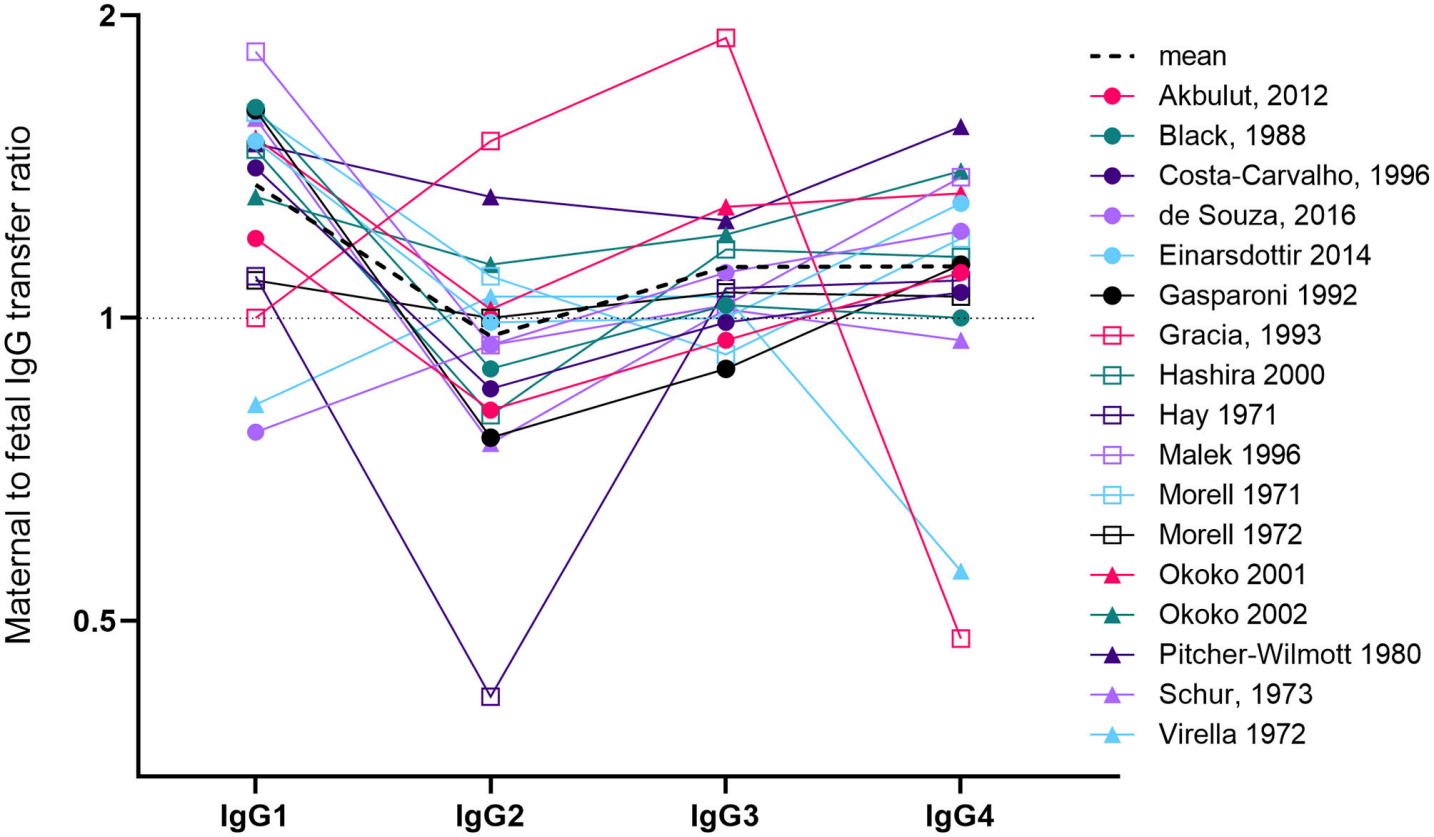
Overview of studies investigating maternal to fetal transfer ratios of IgG subclasses. We identified 17 papers that measured paired maternal and cord levels of all four IgG subclasses (13–22, 24–29). Studies had either employed radioimmunoassay, immunodiffusion, laser nephelometry or ELISA. The line graph represents the mean to fetal transfer ratio on a log(2) axis. Levels >1 (above the dotted line) indicates higher levels in cord blood compared to maternal blood. The dotted black line indicates the mean of the 16 studies. For Malek et al. (29) we only used the values from term pregnancies (37–41weeks). For two studies, we performed ratio calculations based on reported maternal and cord concentrations (21, 29). For two other studies, values were calculated by reading from the published graphs (13, 15). For Costa-Carvalho et al. (13) this required us to read the ratios from the summary graph produced in the review by Palmerio et al. (12). In Hay et al. (28), the maternal levels of IgG1 were the lowest of all the other subclasses and maternal IgG2 was the highest (double the level of IgG1).

IgG is divided into four subclasses, which cross the placenta with differential efficiency. The issue of transplacental transfer of the IgG subclasses is important, as they play diverse roles in immunity, and their production is differentially induced by distinct pathogens and vaccines [reviewed in (11)]. A number of papers and reviews state that the order of transplacental transfer is IgG1>IgG4>IgG3>IgG2, and that IgG2 transfer is “poor”. These usually reference one review (12) that has a graph illustrating data from a single study (13). We have identified 17 papers that have measured maternal and cord levels of the four IgG subclasses (13–29) (Figure 1). Calculating the mean from these papers produces transfer ratios of 1.36 for IgG1, 0.97 for IgG2, 1.12 for IgG3, and 1.12 for IgG4, although the high inter-study variation in both transfer ratios and the transfer hierarchy of the subclasses makes this average an oversimplification. Of the 17 papers, only seven (41%) report the transfer hierarchy of IgG1>IgG4>IgG3>IgG2. The reasons for these varying reports could be due to many differences in study populations including ethnicity, parity, BMI, placental weight, birth weight, gestation at birth, as well as maternal antigen exposure (through vaccination or infection) modifying antibody phenotype (including subclasses, antigen-specificity, and glycosylation).

Many previous studies of placental antibody transfer have been relatively small and often have not interrogated the effect of clinical variables other than gestation at delivery. Later gestation at delivery is associated with an increase in maternal to cord transfer ratios and in concentrations of antibody in cord blood (11, 30–32). However, several additional maternal and fetal factors have the potential to impact on placental antibody subclass transfer. This includes fetal sex, Rhesus status (and associated anti-D antibody administration), maternal vaccination, BMI, parity and ethnicity, among others. It is important to understand the role of these factors when performing maternal vaccination studies in diverse patient populations. We performed IgG subclass analysis on a large number of maternal and cord blood samples, including an assessment of the impact of maternal and fetal factors on IgG subclass levels and transplacental transfer.

## Materials and Methods

### Study Subjects and Sample Collection

Serum samples were obtained from 116 corresponding maternal and–cord blood pairs, as part of two maternal vaccination studies. These studies were approved by Research Ethics Committee (MatImms: REC 13/LO/1712. and COMET: REC 19/YH/0217) and written informed consent was obtained from all participants. Healthy women with singleton pregnancies with no known complications nor chronic or acute diseases were recruited. All pregnancies delivered at term (range 37+3 to 42+3). Detailed clinical data including BMI, parity, age, ethnicity, gestation at delivery, infant sex, birth weight, and maternal vaccination status were recorded. Individualized birthweight centiles (IBC) were calculated using GROW software (Perinatal Institute, version 8.0.4, gestation.net), which controls for maternal height, weight, ethnicity, and parity, and sex and gestation at birth. Serum samples were obtained from mothers near the time of birth (between 1 day before delivery and 3 days after delivery), and from the umbilical cord vein or artery within 1 h of delivery. Where possible, blood was collected separately from both the umbilical vein and the umbilical artery and this was recorded. Blood was collected into serum-separating tubes (BD) and left to clot for a minimum of 15 min before spinning at 1900G for 10 min. Serum was aliquoted and stored at −80°C.

### Immunoglobulin G (IgG) ELISAs

IgGs were measured using human Total IgG, IgG1, IgG2, IgG3, and IgG4 uncoated ELISA kits (Invitrogen) according to the manufacturer's instructions. Absorbance was read at 450 nm using a VersaMax ELISA microplate reader.

### Western Blotting

Washed placental villous tissue was lysed in cold RIPA buffer containing protease and phosphatase inhibitors (cOmplete™ Protease Inhibitor Cocktail and PhosSTOP; Roche), in Lysing Prep D tubes (1.4 mm ceramic spheres), using a FastPrep-24 machine. Following centrifugation, protein supernatants were quantified by BCA assay (Pierce) using a VersaMax Microplate reader (Molecular Devices). Protein was prepared in reducing Laemmli SDS sample buffer (Alfa Aesar) and heated at 95°C for 5 min. Equal amounts of protein (25 μg) were separated on Bis-Tris gels and transferred onto PVDF membrane (iBLOT gel transfer stacks; Invitrogen). Membranes were stained for total protein using the MemCode™ Reversible Protein Stain (Thermo Scientific) following the manufacturer's instructions. This kit utilizes a high affinity protein stain which is compatible with subsequent western blotting. Following staining, membranes were imaged on the ImageQuant camera system (LAS 4000; GE Healthcare) to enable normalization of subsequent specific protein bands. This approach for normalization was employed due to the high variability of common protein loading controls in the placenta (33). After de-staining, membranes were blocked for 1h (5% dry milk, 1% BSA), and probed with 0.1μg/ml anti-FcRn antibody (SC B-8, Santa Cruz Biotechnology) overnight at 4°C. Bound antibody was detected through incubation with 0.125 μg/ml horseradish peroxidase (HRP)–conjugated goat anti-rabbit secondary antibody (Dako). Finally, membranes were developed using ECL Prime Western blotting detection reagent (Amersham) and Hyperfilm (Amersham). FcRn levels relative to total protein was determined by densitometry (ImageJ).

### Statistical Analysis

Statistical analyses were performed in GraphPad Prism v8.0 and STATA v16. IgG concentrations were log transformed (natural Log) prior to analysis to achieve normality. Maternal to fetal transfer ratios were calculated by dividing the IgG concentration in cord blood, by the IgG concentration in paired maternal blood. These ratios were then log(2) transformed prior to analysis. Comparison of transfer ratios was performed using Repeated Measures One Way ANOVA and Holm Sidak's multiple comparisons test. We looked at the effect of the following clinical parameters on antibody transfer: maternal age, maternal body mass index (BMI), rhesus status, ethnicity, parity, vaccination status (tetanus, diphtheria, acellular pertussis and inactivated poliomyelitis [Tdap/IPV] combination vaccination, or seasonal influenza vaccination), gestational age at vaccination, gestational age at delivery, infant sex, and individualized birth ratio (IBC). For ethnicity, we had subjects in 14 groups with small numbers in many; we therefore collapsed the groups into white (white British, white Irish and any other white background; 0) and non-white (black/black British, Asian/Asian British, mixed, and “other”; 1) to enable analysis. For parity, we combined those with parity 1–3 into one “parous” group (coded 1) and compared these to the primiparous group (no previous live births; 0).

Unadjusted and adjusted effects were estimated by simple and multiple linear regression models in Stata v16. Significance was assessed at the 5% level. All covariates that were significant at the 5% level entered the multiple regression models.

## Results

### Maternal and Cord Blood IgG Subclass Profiles and Transfer Ratios

**Figure 2:**
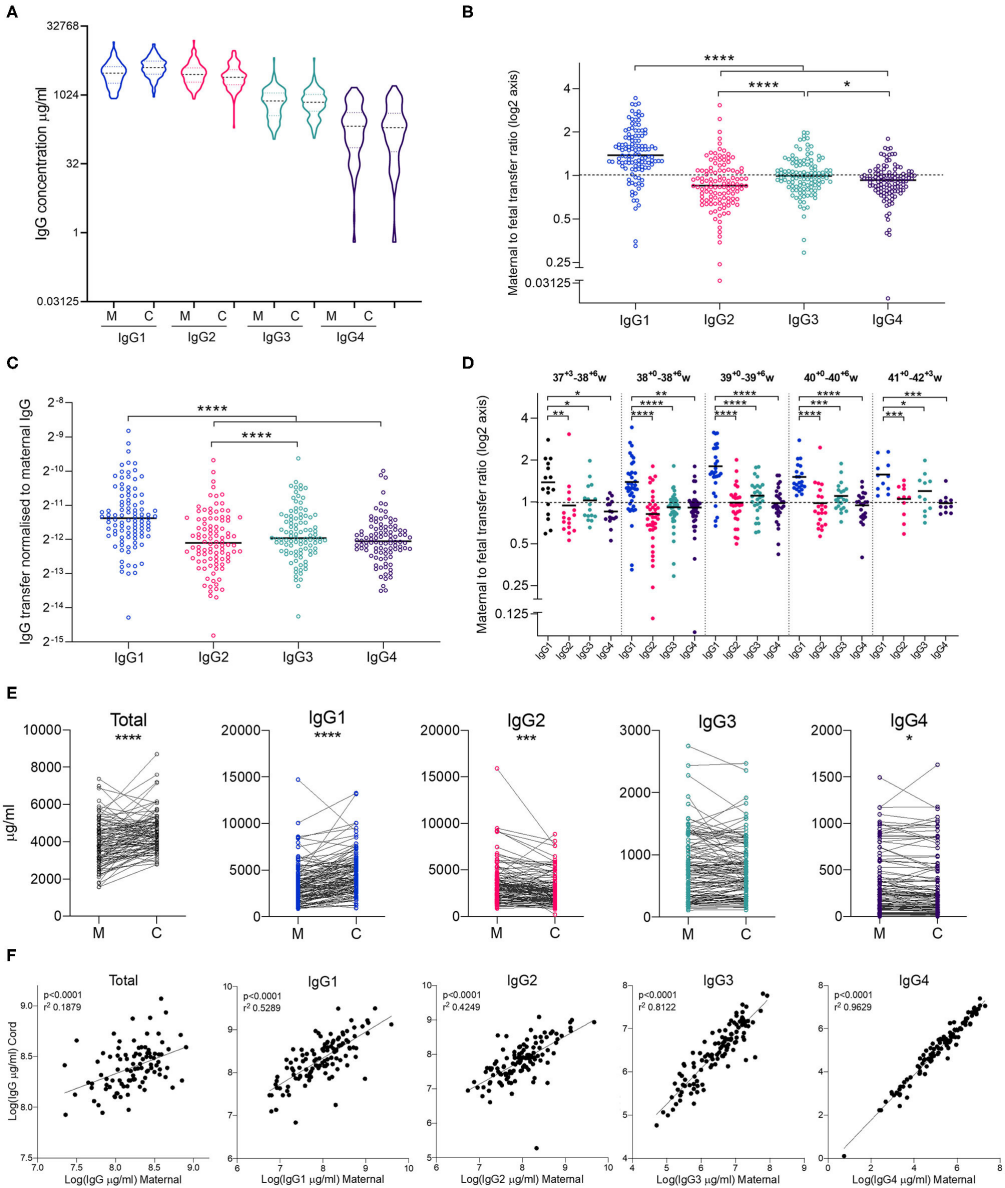
Maternal and cord blood IgG subclass profiles and transfer ratios. **(A)** IgG1-4 concentrations (μg/ml) in maternal (M) and cord (C) blood on a log(2) axis. **(B)** Maternal to fetal transfer ratios of IgG1-4 represented on log(2) scale. Values >1 indicates higher levels in cord blood, indicated by dashed line. Analysis performed on log(2) transformed transfer ratios, using Repeated Measures One Way ANOVA and Holm Sidak's multiple comparisons test. **(C)** Maternal to fetal transfer ratios of IgG1–4 normalized to levels of total IgG in maternal blood as an indicator of transfer efficiency. Analysis by Repeated Measures One Way ANOVA and Holm Sidak's multiple comparisons test. **(D)** Maternal to fetal transfer ratios of IgG1–4 represented on log(2) scale broken down by gestational age at delivery. Values >1 indicates higher levels in cord blood, indicated by dashed line. Analysis performed on log(2) transformed transfer ratios, using Repeated Measures One Way ANOVA and Holm Sidak's multiple comparisons test. Hierarchy of IgG1>IgG3>IgG2>IgG4 for 37–38 weeks and 40+ weeks gestation. Hierarchy of IgG1>IgG3>IgG4>IgG2 for 39 weeks gestation. **(E)** Dot-line plots showing the relationship between IgG concentrations in maternal and cord pairs. Groups compared by paired t test on nLog-transformed data. **(F)** Correlations between maternal and cord IgG concentrations. Lines indicate simple linear regression. nLog values analyzed by Pearson *r-*test. *N* = 114 maternal and cord pairs. ****p < 0.0001, ***p < 0.001, **p < 0.01, *p < 0.05.

Clinical characteristics of the study subjects are shown in Table 1. As mothers were bled at varying times pre-/post-delivery, we checked that this did not impact on maternal IgG concentrations, which would bias the calculation of transfer ratios. Our study subjects were bled 1 day before delivery (*n* = 7), on the day of delivery (*n* = 78), within ½ day of delivery (*n* = 2), within 1 day (*n* = 20), 2 days (*n* = 5), or 3 days (*n* = 3). There was no significant effect of timing of maternal bleed and maternal IgG concentrations (Supplemental Figure 1). Maternal and cord blood exhibited similar IgG subclass profiles, with relative abundance highest for IgG1, followed by IgG2, IgG3, then IgG4 in mothers, as well as their infants (Figure 2A). The maternal to fetal transfer ratio of IgG1 (1.538) was significantly higher than the other subclasses (Figure 2B). IgG3 also exhibited a small, but significantly higher transfer ratio (1.040) than both IgG2 (0.9326) and IgG4 (0.9330). When transfer ratios were normalized to total IgG levels in mothers, as a measure of relative transfer efficiency, a similar pattern of transfer was observed, although IgG3 was no longer significantly higher than IgG4 (Figure 2C). When transfer ratios were compared between pregnancies between 37^+3^ and 42^+3^ weeks gestation, similar relative transfer ratios were also observed, with significantly higher IgG1 transfer than the other subclasses at all gestations (Figure 2D). Paired comparison revealed significantly higher levels of total IgG and IgG1, and significantly lower levels of IgG2 and IgG4 in cord blood serum compared to matched maternal serum (Figure 2E). IgG3 was not significantly different between mothers and infants. The percentage of mother-cord pairs with transfer >1 was 83.6% for IgG1, 49.1% for IgG3, and 31.8% for both IgG2 and IgG4. Correlation plots of maternal and cord IgG levels show a positive correlation between maternal and cord antibody for total IgG and all IgG subclasses. Within the subclasses, the strongest correlation between maternal and cord levels is for IgG4, with the weakest correlation for IgG2 (Figure 2F). Additional descriptive statistics on the transfer of the subclasses is provided in Supplemental Table 1.

**Table 1:** Maternal and infant characteristics.

Maternal age, years (mean and range)	33 (20–45)
Maternal height, cm (mean and range)	165 (135–181)
Maternal weight, kg (mean and range)	67.1 (48.2–111)
BMI (mean and range)	24.6 (17.9–38.9)
**Blood group**	
A (*n* and %)	43 (37%)
B (*n* and %)	14 (12%)
AB (*n* and %)	2 (1.7%)
O (*n* and %)	57 (49%)
Rhesus + (%)	87.9%
Primiparous (%)	58.62%
Non-white ethnicity (%)	41.4%
Tdap vaccinated (%)	61.2%
Gestation at Tdap (weeks)	30.8 (20–39)
Flu vaccinated (%)	50.0%
Gestation at flu (weeks)	24.8 (5–39)
Vaginal delivery (%)	44.0%
Delivery with labor (%)	53.4%
Gestation at delivery (mean and range)	40.1 (37^+3^–42^+3^)
Male sex baby (%)	51.7%
Birth weight, kg (mean and range)	3.45 (2.32–4.45)

### Maternal Antibody Concentrations Are Negatively Correlated With Maternal to Fetal Transfer Ratios of IgG Subclasses

**Figure 3:**
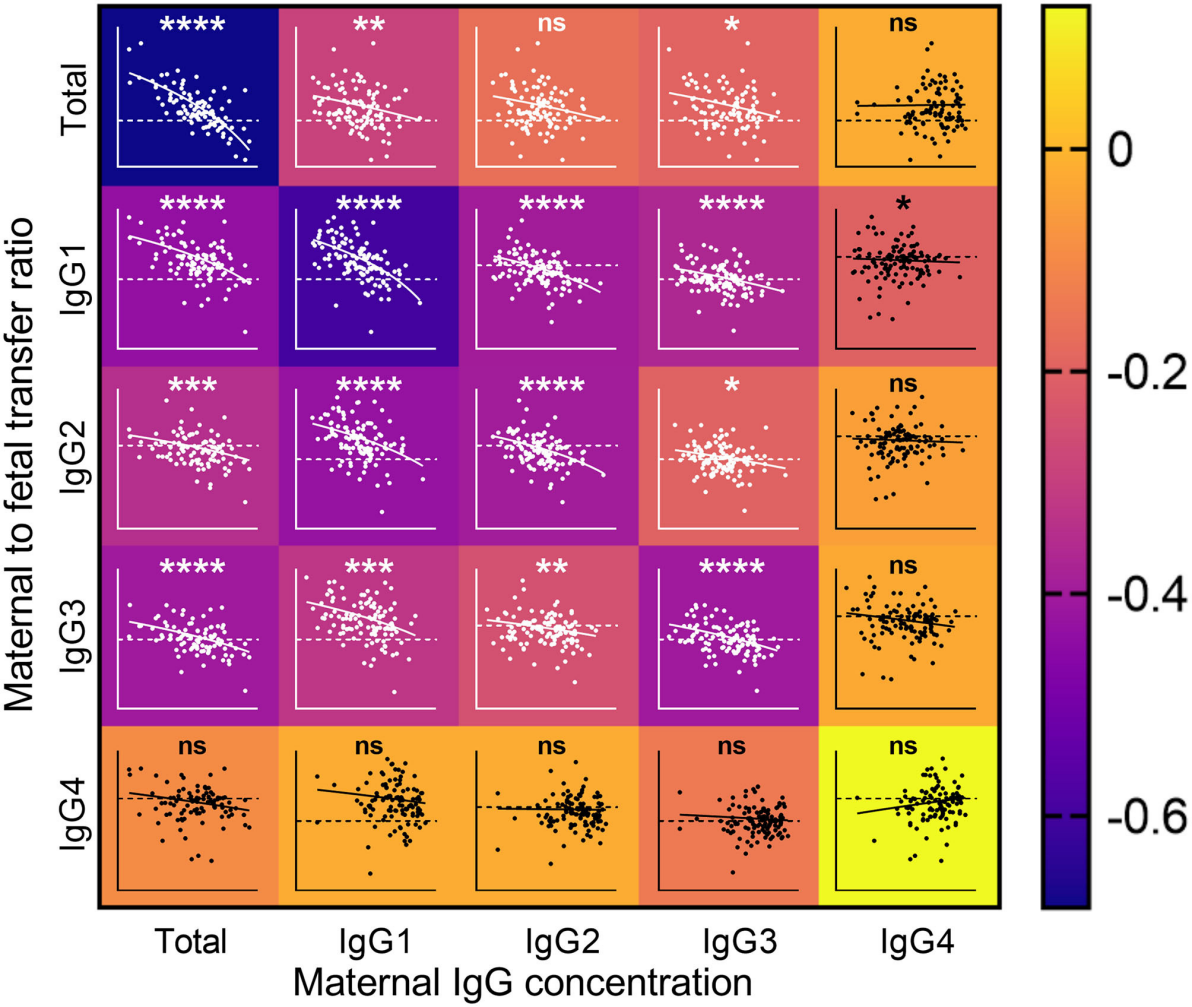
Impact of maternal IgG concentrations on the rate of maternal to fetal IgG transfer. Heatmap with overlaid scatterplots showing the effect of increasing concentrations of maternal IgG (*X* axis) on the transfer rates of total IgG (TIgG) and IgG1–4 from mother to fetus (*Y* axis, Log2 scale). Horizontal dashed line represents equal levels between mother and fetal circulations. Correlations between nlog-transformed maternal IgG concentrations and log(2)-transformed transfer ratios were analyzed by Spearman's rank correlation coefficient. The scale on the right indicates the color associated with the Spearman *r-*values. Inverse correlations are depicted in the darker shades, and weaker and no associations are depicted in lighter shades toward yellow. The lines on the scatterplots indicate simple linear regressions. ****p < 0.0001, ***p < 0.001, **p < 0.01, and *p < 0.05.

As maternal levels of antigen-specific antibodies have been associated with reduced placental transfer ratios (11, 34), we investigated the association between maternal levels of antibody subclasses and transfer ratios (Figure 3). Maternal concentrations of total IgG, IgG1, and IgG3 were negatively associated with the transfer ratios of total IgG. Total IgG, IgG1, IgG2, and IgG3 were negatively associated with transfer of IgG1–IgG3. Maternal concentrations of IgG4 were only negatively associated with the transfer ratios of IgG1.

### Maternal to Fetal Transfer Ratios Are Largely Unaffected by Sampling of the Umbilical Artery or Umbilical Vein

**Figure 4:**
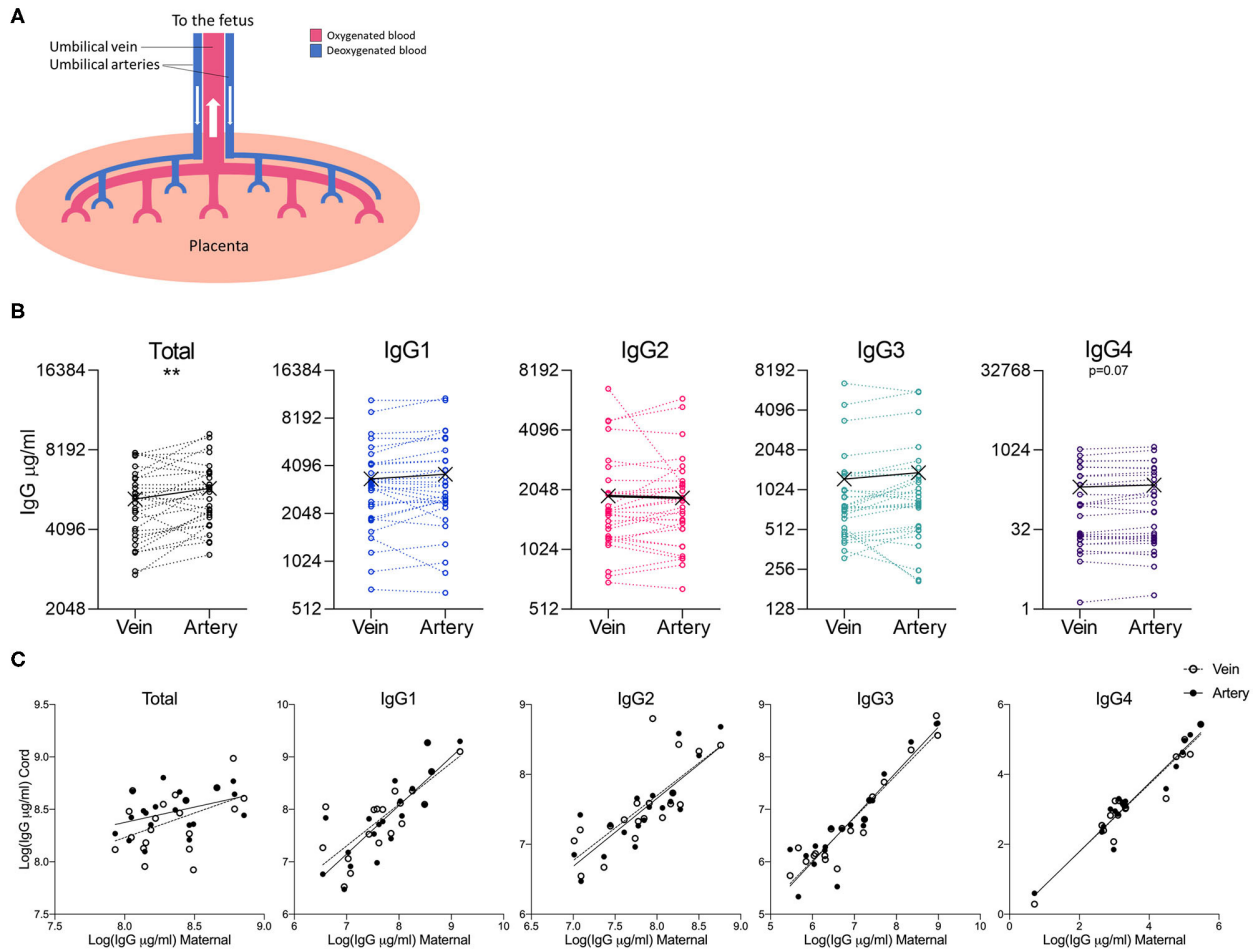
Comparison of IgG subclasses in umbilical cord vein and arterial blood. **(A)** Illustration of the placental circulation, showing the umbilical arteries bringing deoxygenated blood from the placenta to the fetus, and oxygenated blood returning to the fetus from the placenta via the umbilical vein. **(B)** Dot-line plots showing the relationship between IgG concentrations in paired umbilical cord vein and artery serum samples (*n* = 30). Groups compared by paired *t-*test on nLog-transformed data. **p < 0.01 **(C)** Correlations between maternal and cord IgG concentrations. Lines indicate simple linear regression. nLog values analyzed by Pearson *r-*test. *N* = 30 matched umbilical vein/umbilical artery serum pairs and 20 matched maternal/umbilical vein/umbilical artery serum trios.

As the umbilical vein represents oxygenated blood flowing from the placenta, and the umbilical arteries represent deoxygenated blood flowing from the fetus (Figure 4A), we investigated whether any differences could be observed in blood sampled from these two vessels. Although total IgG levels were significantly higher in arterial blood, this difference was not borne out in the analysis of the IgG subclasses (Figure 4B). Both maternal: umbilical vein IgG levels, and maternal: umbilical artery IgG levels were positively correlated (Figure 4C).

### Correlation Between Placental FcRn Expression and Transfer of IgG Subclasses

**Figure 5:**
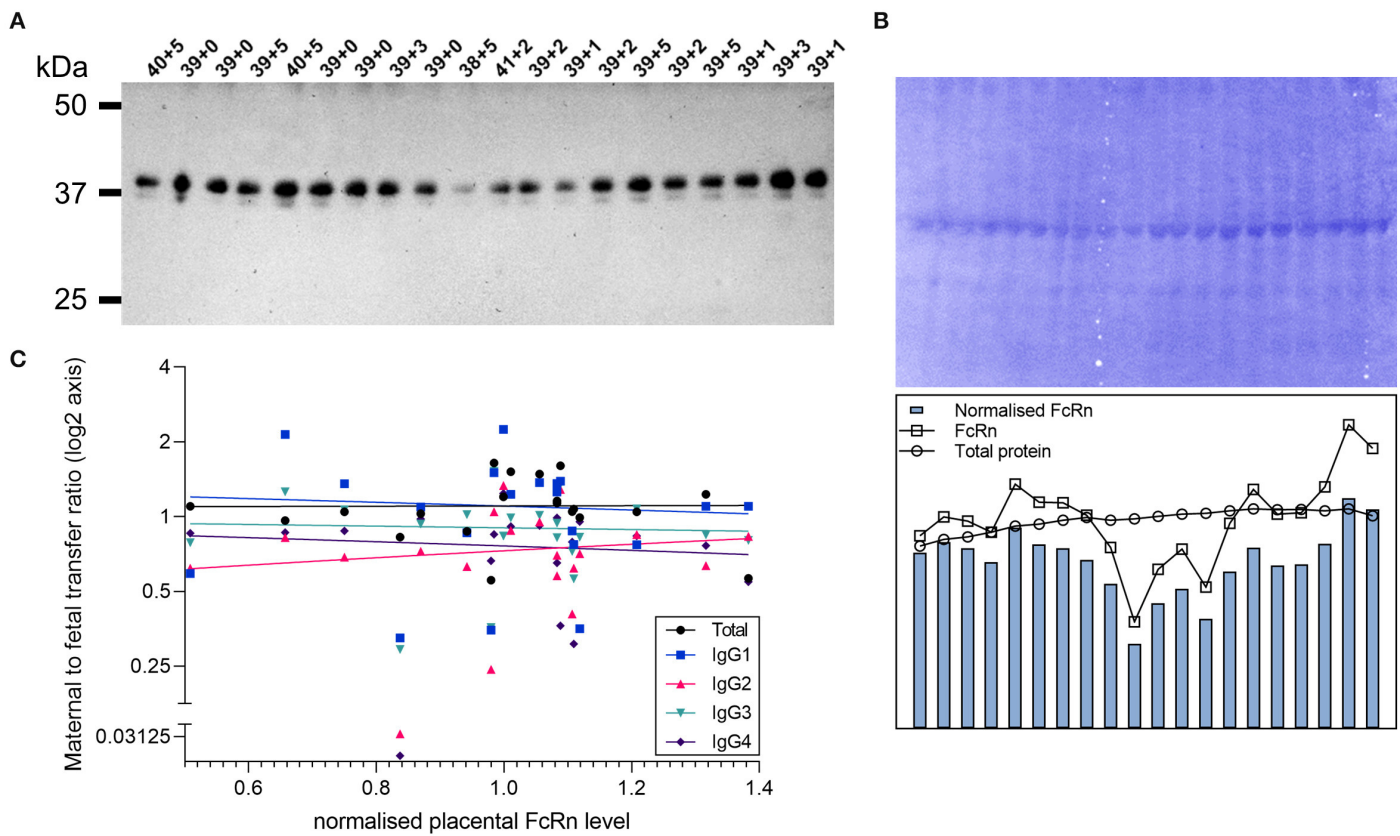
Correlation of placental FcRn levels with transfer of IgG subclasses. **(A)** Western blot for FcRn in lysates of placental villous tissue. Proteins were prepared under reducing denaturing conditions. Top labels indicate gestational age of the sample in weeks+days. **(B)** Total protein stain (left panel) and densitometry analysis (right panel). **(C)** Correlation between placenta FcRn protein levels (normalized to total protein stain) and maternal to fetal IgG transfer ratios (log(2) transformed) analyzed by Spearman's rank correlation coefficient. All correlations non-significant at *p* > 0.05 level (Total IgG *p* = 0.51, Spearman *r* 0.156; IgG1 *p* = 0.80, *r* = −0.062; IgG2 *p* = 0.54, *r* = 0.146; IgG3 *p* = 0.67, *r* = −0.101; IgG4 *p* = 0.26, *r* = −0.266). *N* = 20.

Transplacental antibody transfer across the synctytiotrophoblast layer of the placenta is mediated through interaction with the neonatal Fc receptor [FcRn; reviewed in (10)]. To investigate whether inter-individual differences in IgG subclass transfer are due to differences in FcRn levels in the placenta, western blotting of paired placental samples was performed (Figure 5). With the set of paired samples available (*n* = 20), no association was detected between placental FcRn levels and the transfer ratio of total IgG nor subclasses 1–4 (Figure 5C).

### Investigation of Clinical Variables Associated With Levels and Transfer of IgG: Unadjusted Effects

Unadjusted analyses were performed to explore relationships between clinical variables and maternal and cord IgG concentrations, as well as the maternal to fetal transfer of IgG subclasses (Tables 2–4). Increased maternal age was associated with lower total IgG in mothers (*p* = 0.042; Table 2), but had no effect on cord levels (Table 3) nor transfer (Table 4). Non-white women had higher levels of total IgG, IgG2, and IgG3 (*p* = 0.034, 0.019, and 0.024; Table 2). There was no corresponding increase in infant IgG levels, and the calculated transfer ratios of IgG3 in these pregnancies was significantly lower (*p* = 0.004; Table 4). Parous women had higher levels of total IgG (*p* = 0.004) and IgG1 (*p* = 0.034) compared to primiparous. The transfer ratios of total, IgG1 and IgG3 in these pregnancies was significantly lower (*p* = 0.023, 0.001, and 0.001; Table 4), resulting in no corresponding increase in infant IgG levels (Table 3). Although maternal rhesus status had no effect on maternal IgG levels, rhesus negative pregnancies were associated with lower transfer of IgG2 (*p* = 0.048) and a corresponding lower level of IgG2 in cord blood (*p* = 0.047) compared to rhesus positive pregnancies.

**Table 2:** Unadjusted effects of clinical parameters on maternal antibody concentrations using simple linear regression based on *n* = 106 for total IgG and *n* = 116 for IgG subclasses.

**Variable**	**Antibody**	**Coefficient**	**Standard error**	***p*-value**	**95% CI**
Maternal age (years)	Total	−0.014	0.007	**0.042**	−0.027, −0.001
	IgG1	−0.013	0.012	0.275	−0.037, 0.011
	IgG2	−0.007	0.011	0.546	−0.029, 0.015
	IgG3	0.012	0.015	0.401	−0.017, 0.041
	IgG4	−0.020	0.029	0.484	−0.077, 0.037
Maternal BMI	Total	0.008	0.008	0.305	−0.007, 0.023
	IgG1	−0.004	0.014	0.759	−0.032, 0.023
	IgG2	−0.003	0.013	0.815	−0.028, 0.022
	IgG3	0.015	0.017	0.368	−0.018, 0.048
	IgG4	−0.007	0.033	0.826	−0.073, 0.058
Maternal ethnicity (white = 0; non-white = 1)	Total	0.139	0.065	**0.034**	0.011, 0.268
	IgG1	0.170	0.114	0.141	−0.057, 0.396
	IgG2	0.247	0.104	**0.019**	0.041, 0.452
	IgG3	0.314	0.138	**0.024**	0.042, 0.587
	IgG4	−0.065	0.277	0.815	−0.614, 0.484
Parity (primiparous = 0; parous = 1)	Total	0.188	0.063	**0.004**	0.062, 0.314
	IgG1	0.243	0.113	**0.034**	0.019, 0.467
	IgG2	0.187	0.105	0.077	−0.020, 0.394
	IgG3	0.263	0.139	0.061	−0.012, 0.537
	IgG4	0.158	0.277	0.570	−0.390, 0.706
Rhesus status (negative = 0; positive = 1)	Total	0.078	0.111	0.486	−0.142, 0.298
	IgG1	0.112	0.174	0.523	−0.233, 0.457
	IgG2	0.072	0.161	0.655	−0.246, 0.390
	IgG3	0.204	0.212	0.339	−0.216, 0.624
	IgG4	−0.441	0.418	0.292	−1.267, 0.385
Gestation at delivery (weeks)	Total	−0.068	0.028	**0.018**	−0.124, −0.012
	IgG1	0.026	0.051	0.605	−0.074, 0.127
	IgG2	−0.013	0.047	0.779	−0.106, 0.079
	IgG3	−0.010	0.062	0.874	−0.132, 0.113
	IgG4	0.361	0.117	**0.003**	0.129, 0.593
Sex of baby (male = 0; female = 1)	Total	−0.018	0.065	0.780	−0.148, 0.111
	IgG1	0.033	0.114	0.775	−0.193, 0.258
	IgG2	0.093	0.104	0.373	−0.113, 0.300
	IgG3	−0.138	0.138	0.321	−0.411, 0.136
	IgG4	0.248	0.272	0.365	−0.291, 0.787
Individualized birth ratio (IBC)	Total	−0.002	0.001	0.075	−0.004, 0.001
	IgG1	0.0001	0.002	0.942	−0.004, 0.004
	IgG2	−0.002	0.002	0.355	−0.005, 0.002
	IgG3	0.003	0.002	0.263	−0.002, 0.008
	IgG4	−0.003	0.005	0.544	−0.013, 0.007
Tdap maternal vaccination status (non-vacc = 0; vacc = 1)	Total	0.003	0.067	0.966	−0.126, 0.135
	IgG1	0.180	0.115	0.121	−0.049, 0.409
	IgG2	0.035	0.107	0.742	−0.177, 0.248
	IgG3	0.146	0.142	0.305	−0.135, 0.426
	IgG4	−0.031	0.280	0.911	−0.586, 0.523
Tdap maternal vaccination timing (gestational weeks)	Total	−0.008	0.014	0.593	−0.036, 0.021
	IgG1	−0.022	0.023	0.328	−0.068, 0.023
	IgG2	−0.025	0.020	0.226	−0.066, 0.016
	IgG3	0.003	0.027	0.919	−0.052, 0.058
	IgG4	0.012	0.052	0.823	−0.093, 0.116
Flu maternal vaccination status (non-vacc = 0; vacc = 1)	Total	0.032	0.065	0.621	−0.097, 0.161
	IgG1	0.183	0.112	0.107	−0.040, 0.405
	IgG2	−0.038	0.105	0.718	−0.245, 0.169
	IgG3	0.090	0.138	0.519	−0.185, 0.364
	IgG4	0.563	0.268	**0.038**	0.033, 1.094
Flu maternal vaccination timing (gestational weeks)	Total	0.015	0.006	**0.012**	0.003, 0.026
	IgG1	0.004	0.010	0.668	−0.016, 0.025
	IgG2	0.017	0.009	0.057	−0.001, 0.035
	IgG3	0.006	0.013	0.650	−0.020, 0.032
	IgG4	−0.001	0.023	0.982	−0.046, 0.045

*Bold values indicate p value < 0.05*.

**Table 3:** Unadjusted effects of clinical parameters on umbilical cord antibody concentrations using simple linear regression based on *n* = 106 for total IgG and *n* = 116 for IgG subclasses.

	**Antibody**	**Coefficient**	**Standard error**	***p-*value**	**95% CI**
Maternal age (years)	Total	−0.006	0.005	0.234	−0.016, 0.004
	IgG1	−0.014	0.010	0.162	−0.034, 0.006
	IgG2	−0.006	0.012	0.622	−0.029, 0.017
	IgG3	0.013	0.013	0.312	−0.013, 0.040
	IgG4	−0.018	0.032	0.577	−0.081, 0.045
Maternal BMI	Total	0.004	0.006	0.443	−0.007, 0.015
	IgG1	−0.009	0.011	0.445	−0.031, 0.014
	IgG2	−0.002	0.013	0.876	−0.028, 0.024
	IgG3	0.006	0.015	0.685	−0.024, 0.037
	IgG4	−0.021	0.036	0.560	−0.093, 0.051
Maternal ethnicity (white = 0; non-white = 1)	Total	0.054	0.047	0.255	−0.040, 0.148
	IgG1	0.025	0.096	0.792	−0.165, 0.215
	IgG2	0.163	0.111	0.142	−0.055, 0.382
	IgG3	0.152	0.129	0.240	−0.103, 0.408
	IgG4	0.008	0.304	0.978	−0.059, 0.611
Parity (primiparous = 0; parous = 1)	Total	0.056	0.047	0.236	−0.037, 0.149
	IgG1	−0.018	0.096	0.855	−0.208, 0.172
	IgG2	0.031	0.112	0.784	−0.190, 0.252
	IgG3	0.080	0.130	0.539	−0.177, 0.337
	IgG4	−0.049	0.304	0.873	−0.651, 0.553
Rhesus status (negative = 0; positive = 1)	Total	0.076	0.079	0.338	−0.081, 0.234
	IgG1	0.177	0.144	0.223	−0.109, 0.462
	IgG2	0.334	0.166	**0.047**	0.005, 0.662
	IgG3	0.275	0.194	0.161	−0.111, 0.660
	IgG4	−0.140	0.459	0.762	−1.049, 0.770
Gestation at delivery (weeks)	Total	0.018	0.021	0.375	−0.023, 0.060
	IgG1	0.104	0.041	**0.013**	0.022, 0.185
	IgG2	0.059	0.049	0.229	−0.038, 0.156
	IgG3	0.061	0.057	0.283	−0.051, 0.174
	IgG4	0.429	0.128	**0.001**	0.176, 0.681
Sex of baby (male = 0; female = 1)	Total	0.034	0.047	0.469	−0.059, 0.127
	IgG1	0.000	0.096	0.999	−0.187, 0.187
	IgG2	0.022	0.110	0.838	−0.195, 0.240
	IgG3	−0.137	0.127	0.283	−0.390,0.115
	IgG4	0.179	0.299	0.550	−0.413, 0.772
Individualized birth ratio (IBC)	Total	−0.002	0.001	**0.004**	−0.004, −0.001
	IgG1	−0.003	0.002	0.099	−0.006, 0.001
	IgG2	−0.004	0.002	**0.043**	−0.008, −0.000
	IgG3	0.002	0.002	0.294	−0.002,0.007
	IgG4	−0.008	0.005	0.139	−0.018, 0.003
Tdap maternal vaccination status (non-vacc = 0; vacc = 1)	Total	0.047	0.048	0.332	−0.048, 0.141
	IgG1	0.076	0.097	0.433	−0.116, 0.268
	IgG2	−0.055	0.113	0.626	−0.278, 0.168
	IgG3	0.165	0.130	0.207	−0.093, 0.423
	IgG4	−0.140	0.307	0.649	−0.748, 0.468
Tdap maternal vaccination timing (gestational weeks)	Total	0.016	0.009	0.079	−0.002, 0.034
	IgG1	0.031	0.019	0.115	−0.008, 0.069
	IgG2	0.024	0.023	0.296	−0.022, 0.070
	IgG3	0.033	0.025	0.199	−0.018, 0.083
	IgG4	0.090	0.059	0.133	−0.028, 0.208
Flu maternal vaccination status (non-vacc = 0; vacc = 1)	Total	0.005	0.047	0.912	−0.087, 0.098
	IgG1	0.073	0.094	0.441	−0.114, 0.260
	IgG2	0.002	0.110	0.989	−0.216, 0.219
	IgG3	0.088	0.128	0.489	−0.164, 0.341
	IgG4	0.696	0.292	**0.019**	0.117, 1.275
Flu maternal vaccination timing (gestational weeks)	Total	0.009	0.004	0.056	−0.000, 0.018
	IgG1	0.001	0.009	0.948	−0.017, 0.018
	IgG2	0.005	0.009	0.619	−0.014, 0.023
	IgG3	−0.006	0.012	0.631	−0.029, 0.018
	IgG4	−0.002	0.024	0.934	−0.051, 0.047

*Bold values indicate p value < 0.05*.

**Table 4:** Unadjusted effects of clinical parameters on log(2) maternal to fetal IgG transfer ratios using simple linear regression based on *n* = 106 for total IgG and *n* = 116 for IgG subclasses.

**Variable**	**Antibody**	**Coefficient**	**Standard error**	***p-*value**	**95% CI**
Maternal age (years)	Total	0.012	0.009	0.189	−0.006, 0.029
	IgG1	−0.001	0.012	0.920	−0.025, 0.022
	IgG2	0.001	0.013	0.918	−0.025, 0.028
	IgG3	0.002	0.009	0.825	−0.015, 0.019
	IgG4	0.004	0.018	0.835	−0.031, 0.039
Maternal BMI	Total	−0.005	0.010	0.593	−0.025, 0.014
	IgG1	−0.007	0.013	0.628	−0.033, 0.020
	IgG2	0.001	0.015	0.932	−0.029, 0.031
	IgG3	−0.013	0.010	0.200	−0.032, 0.007
	IgG4	−0.020	0.020	0.319	−0.060, 0.020
Maternal ethnicity (white = 0; non–white = 1)	Total	−0.123	0.084	0.147	−0.289, 0.044
	IgG1	−0.208	0.111	0.064	−0.429, 0.012
	IgG2	−0.120	0.127	0.344	−0.371, 0.131
	IgG3	−0.234	0.081	**0.004**	−0.394, −0.074
	IgG4	0.106	0.168	0.530	−0.228, 0.440
Parity (primiparous = 0; parous = 1)	Total	−0.190	0.083	**0.023**	−0.354, −0.026
	IgG1	−0.376	0.107	**0.001**	−0.588, −0.163
	IgG2	−0.226	0.125	0.074	−0.474, 0.022
	IgG3	−0.264	0.080	**0.001**	−0.422, −0.105
	IgG4	−0.298	0.166	0.076	−0.627, 0.032
Rhesus status (negative = 0; positive = 1)	Total	−0.002	0.143	0.990	−0.285, 0.281
	IgG1	0.094	0.171	0.583	−0.244, 0.432
	IgG2	0.378	0.189	**0.048**	0.003, 0.752
	IgG3	0.103	0.126	0.417	−0.147, 0.352
	IgG4	0.435	0.252	0.087	−0.064, 0.934
Gestation at delivery (weeks)	Total	0.110	0.035	**0.002**	0.041, 0.179
	IgG1	0.099	0.048	**0.040**	0.005, 0.193
	IgG2	0.104	0.054	0.055	−0.002, 0.210
	IgG3	0.086	0.035	**0.015**	0.017, 0.155
	IgG4	0.084	0.072	0.245	−0.058, 0.226
Sex of baby (male = 0; female = 1)	Total	0.075	0.083	0.368	−0.090, 0.241
	IgG1	−0.047	0.111	0.675	−0.267, 0.174
	IgG2	−0.102	0.125	0.415	−0.350, 0.145
	IgG3	0.001	0.082	0.994	−0.162, 0.164
	IgG4	−0.099	0.166	0.554	−0.428, 0.230
Individualized birth ratio (IBC)	Total	−0.000	0.001	0.764	−0.003, 0.003
	IgG1	−0.004	0.002	**0.032**	−0.008, −0.000
	IgG2	−0.003	0.002	0.151	−0.008, 0.001
	IgG3	−0.001	0.001	0.716	−0.003, 0.002
	IgG4	−0.007	0.003	**0.015**	−0.013, −0.001
Tdap maternal vaccination status (non-vacc = 0; vacc = 1)	Total	0.063	0.085	0.462	−0.106, 0.232
	IgG1	−0.150	0.113	0.188	−0.375, 0.074
	IgG2	−0.131	0.128	0.310	−0.384, 0.123
	IgG3	0.028	0.084	0.743	−0.139, 0.195
	IgG4	−0.157	0.170	0.358	−0.494, 0.180
Tdap maternal vaccination timing (gestational weeks)	Total	0.034	0.018	0.063	−0.002, 0.069
	IgG1	0.076	0.021	**0.001**	0.034, 0.118
	IgG2	0.071	0.025	**0.007**	0.020, 0.122
	IgG3	0.043	0.015	**0.006**	0.013, 0.073
	IgG4	0.112	0.038	**0.004**	0.037, 0.187
Flu maternal vaccination status (non-vacc = 0; vacc = 1)	Total	−0.039	0.083	0.640	−0.204, 0.126
	IgG1	−0.158	0.110	0.154	−0.377, 0.060
	IgG2	0.057	0.125	0.650	−0.191, 0.305
	IgG3	−0.002	0.082	0.985	−0.165, 0.161
	IgG4	0.192	0.165	0.247	−0.135, 0.519
Flu maternal vaccination timing (gestational weeks)	Total	−0.009	0.008	0.270	−0.024, 0.007
	IgG1	−0.005	0.011	0.619	−0.027, 0.017
	IgG2	−0.018	0.010	0.076	−0.039, 0.002
	IgG3	−0.017	0.006	**0.013**	−0.030, −0.004
	IgG4	−0.002	0.007	0.750	−0.016, 0.012

*Bold values indicate p value < 0.05*.

Later gestation at delivery was associated with lower maternal levels of total IgG (*p* = 0.018) and higher IgG4 (*p* = 0.003). Transfer ratios were higher with later gestation at delivery for total IgG, IgG1, and IgG3 (*p* = 0.002, 0.04, and 0.015; Table 4). The outcome of later gestation at delivery was higher neonatal levels of IgG1 and IgG4 (*p* = 0.013, 0.001; Table 3). Neonates with higher birth centiles had lower levels of total IgG and IgG2 (*p* = 0.004, 0.042; Table 3). Pregnancies with higher centile infants also had lower maternal to fetal transfer of IgG1 (*p* = 0.032) and IgG4 (*p* = 0.015; Table 4). Maternal BMI and sex of the neonate had no impact on any variables.

### Impact of Maternal Tdap/IPV and Influenza Vaccination on IgG Subclass Levels and Transfer

Sixty-one percent of our cohort received Tdap vaccination in pregnancy, and 50% received seasonal influenza vaccine (Table 1). Tdap vaccination status had no effect on maternal or cord IgG concentrations nor IgG transfer. Influenza vaccination was associated with higher IgG4 in mothers (*p* = 0.038; Table 2) and neonates (*p* = 0.019; Table 3), with no impact on transfer ratios (Table 4).

**Figure 6:**
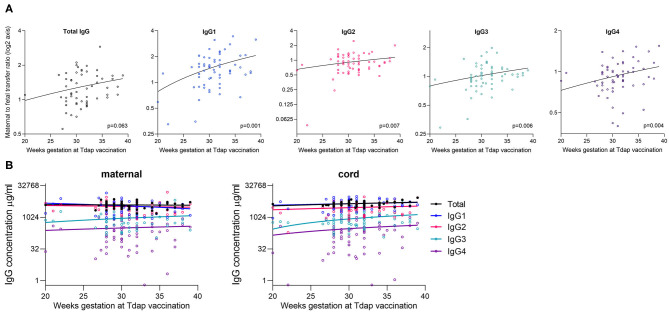
Correlation between gestation at Tdap vaccination and placental transfer of total IgG and IgG subclasses. **(A)** Correlation between the gestation that pregnant women received the Tdap vaccine and the maternal to fetal IgG transfer ratio for total IgG and IgG subclasses [log(2) *Y*-axis]. Analysis was performed on log(2) transformed ratios by Spearman's rank correlation coefficient, and resultant *p* values are stated in the bottom right corner of each plot. Coefficients: Total IgG: 0.242, IgG1: 0.316, IgG2: 0.171, IgG3: 0.353, and IgG4: 0.316. **(B)** Correlation between the gestation at vaccination and the antibody concentration in maternal blood and cord blood at birth. Analysis performed on log-transformed data by Spearman's rank correlation coefficient. All correlations non-significant at *p* > 0.05 level. Lines indicate simple linear regression. Coefficients for maternal IgG: Total IgG: −0.044; *p* = 0.752, IgG1: −0.145; *p* = 0.262, IgG2: −0.180; *p* = 0.161, IgG3: 0.013; *p* = 0.919, IgG4: 0.029; *p* = 0.823. Coefficients for cord IgG: Total IgG: 0.235; *p* = 0.079, IgG1: 0.197; *p* = 0.125, IgG2: 0.134; *p* = 0.296, IgG3: 0.164; *p* = 0.199, and IgG4: 0.191; *p* = 0.133. *N* = 63.

The gestation at Tdap vaccination was positively correlated with maternal to fetal transfer for IgG1 (*p* = 0.001), IgG2 (*p* = 0.007), IgG3 (*p* = 0.006), and IgG4 (*p* = 0.004; Table 4, Figure 6A). There was no association between timing of Tdap and maternal nor cord IgG concentrations (Tables 2, 3, Figure 6B). Later gestation at influenza vaccination was associated with higher levels of total IgG in mums (*p* = 0.012; Table 2), but had no effect on neonatal levels nor transfer. The transfer ratio of IgG3 was lower with later gestation at vaccination (*p* = 0.013; Table 4).

### Adjusted Effects Associated With Transfer of IgG Subclasses

We next performed multiple regression analyses that included the variables that were found to be significant at the 5% level in our unadjusted analyses (Tables 2–4).

In the adjusted analysis, levels of total IgG in maternal serum was associated with both parity and maternal age, but not with gestation at delivery nor ethnicity (Table 5). Parous women had higher levels of total IgG than primiparous women (*p* = 0.015), and older women had lower levels of total IgG (*p* = 0.018), after adjusting for gestation at delivery and for ethnicity. Parous pregnancies had lower transfer ratios of IgG1 (*p* = 0.006), after adjusting for gestation at delivery and birth centiles. Later gestation at delivery was associated with higher transfer ratios of total IgG and IgG3 (*p* = 0.008). IgG3 transfer was also significantly lower in pregnancies of non-white women (*p* = 0.012).

**Table 5:** Adjusted effects of clinical parameters on maternal and cord antibody levels, and transfer ratios using multiple linear regression based on *n* = 106 for total IgG and *n* = 116 for IgG subclasses.

**Outcome**	**Covariates**	**Coefficient**	**Standard error**	***p-*value**	**95% CI**
**Maternal IgG**					
Total IgG	Parity	0.166	0.067	**0.015**	0.033, 0.297
	Maternal age	−0.016	0.007	**0.018**	−0.027, −0.003
	Gestation at delivery	−0.128	0.080	0.110	−0.286, 0.030
	Ethnicity	0.054	0.066	0.413	−0.076, 0.185
**Transfer ratio**					
Total IgG	Gestation at delivery	0.096	0.036	**0.008**	0.025, 0.166
	Parity	−0.135	0.083	0.105	−0.299, 0.029
IgG1	Parity	−0.312	0.110	**0.006**	−0.530, −0.093
	IBC	−0.003	0.002	0.115	−0.007, 0.001
	Gestation at delivery	0.064	0.047	0.170	−0.028, 0.157
IgG3	Ethnicity	−0.207	0.081	**0.012**	−0.366, −0.047
	Gestation at delivery	0.072	0.035	**0.041**	0.003, 0.140

*Covariates: Parity: primiparous = 0, parous = 1; Maternal age: years; Gestation at delivery: weeks; Ethnicity: white = 0, non-white = 1*.

*IBC, individualized birth centile*.

We investigated the association between the clinical variables in these models. Parity was associated with gestation at birth (*p* = 0.019), with the individualized birth weight centile (IBC; *p* = 0.048), and with ethnicity (*p* = 0.003). Primiparous women were more likely to be white (log-odds: 1.216; 95% CI: 0.408, 2.025), had a higher mean gestation at delivery (coef: −0.497; 95% CI: −0.908, −0.085), and their offspring had a lower mean birth centile (coef: 10.423; 95% CI: 0.080, 20.766) compared to parous. There was no association between maternal age and parity, ethnicity nor gestation at delivery. Ethnicity was also associated with gestation at delivery (*p* = 0.049); non-white women had a lower mean gestation at birth compared to white women (coef: −0.417; 95% CI: −0.832, −0.003).

## Discussion

We have performed a comprehensive analysis of maternal and cord levels of the IgG subclasses and transplacental transfer in a UK pregnancy cohort, and investigated their association with maternal and fetal factors.

As previously observed, maternal concentrations of total IgG and IgG subclasses were positively associated with umbilical cord concentrations (Figure 2F) and negatively associated with the maternal to fetal transfer ratios (Figure 3). As has been previously well established, we found higher levels of total IgG and IgG1 in cord blood compared to maternal blood, reflected in a mean transfer ratio of 1.5 for IgG1. The transfer ratio of the other subclasses was near to 1. In our study population the transfer efficiency of the subclasses was IgG1>IgG3>IgG4=IgG2 (Figure 2B). The transfer ratio of IgG1 was significantly higher than the ratios of the other subclasses at all gestations studied (between 37 weeks and 42 weeks; Figure 2D), suggesting that delivery post-dates does not affect the relative maternal/cord ratios of IgG subclasses.

Maternal antibodies may compete for transplacental transfer through their competition for placental FcRn, which binds IgG in a 2:1 stoichiometry (35). Several studies have shown that maternal total and antigen-specific IgG concentrations are inversely correlated with maternal/cord ratios of total and antigen-specific IgG (11, 17, 34, 36), supporting the concept that antibody does not passively diffuse across the placenta to reach equilibrium between maternal and fetal circulations, but requires interaction with FcRn (and other potential Fc receptors) for its transfer. There have been fewer studies of the association between maternal levels of the IgG subclasses and transplacental transfer. One study of placental malaria and maternal hypergammaglobulinemia found an association between maternal IgG levels >15g/L IgG and reduced transfer of IgG1 and IgG2, with no effect on IgG3 and IgG4, compared to maternal levels <15g/L (17). Our analysis performing correlations on continuous data found a negative correlation between maternal total IgG levels and the transfer ratios of total IgG, IgG1, IgG2, and IgG3 subclasses in healthy pregnancy. We also found that maternal concentrations of IgG1, IgG2, and IgG3 were negatively correlated with transfer ratios of total IgG and IgG1–3. This suggests that the higher abundance IgG1, IgG2, and IgG3 may all compete with their respective, and other subclasses, for transfer across the placenta. Importantly, it must be noted that within maternal blood, the concentrations of IgG2, IgG3, and IgG4 are all correlated with IgG1. Thus, the effects observed cannot be necessarily be attributed specifically to a certain subclass, but could be driven by IgG1, which is highest in abundance and transferred with the highest efficiency. With that said, increased maternal IgG4 levels had limited correlation with IgG transfer, and transfer of IgG4 was not correlated with maternal levels of any antibody, even though maternal IgG4 levels are positively correlated with maternal IgG1. Furthermore, IgG4 had the strongest correlation between maternal and cord IgG concentrations, which altogether suggests that the transfer of this subclass is least affected by changes in amounts of antibody in the mother. The clinical significance of this phenomenon on passive immunity in the neonate is likely to lie in the potential impact on the transfer of antibodies against specific pathogens, which should be explored.

It is important to note that this commonly utilized approach of employing maternal/cord IgG ratios as an indicator of transplacental transfer efficiency has its limitations. The relative abundance between paired maternal-cord samples is not necessarily a true reflection of transfer efficiency as this could be biased by maternal concentrations, as well as the levels of IgG recycling in fetal and maternal circulations. To partly address this, we have shown that normalization of transfer ratios to total IgG levels in maternal blood show the same pattern of differential transfer of the IgG subclasses (Figure 2C). We also show a similar pattern of relative transfer ratios between 37 and 42 weeks gestation. *In vitro* competition assays in human trophoblast or non-human primate *in vivo* experiments with spike-in of IgG subclasses are required to fully understand the competition for transplacental transfer between the IgG subclasses. Nevertheless, our data appears to support the current consensus that although higher IgG levels in the mother will result in higher IgG levels in the offspring, the relative transfer efficiency across the placenta can be reduced when maternal concentrations are elevated.

Given the limited capability for IgG production in the fetus, the majority of antibody in the fetal circulation is maternal in origin. We hypothesized that IgG levels in the umbilical arteries (bringing deoxygenated blood from the fetus) would be higher than levels in the umbilical vein (bringing freshly oxygenated blood from the placenta). In contrast however, we found no significant difference in the abundance of the IgG subclasses, and only a slightly but significantly higher level of total IgG in the umbilical artery. This was a surprising finding and could suggest that there is greater hemodilution in venous blood compared to arterial blood, or it could reflect efficient total antibody recycling in the fetal circulation leading to higher levels in blood coming from the fetus. No other study has investigated this to our knowledge. It could be of interest to investigate FcRn levels in the venous and arterial endothelium of the umbilical cord and in the placental vasculature, as this is involved in antibody recycling, thereby increasing half-life. It would also be of interest to determine whether there are differences in the levels of antigen-specific antibodies between venous and arterial umbilical blood. This finding suggests that it is important that maternal vaccination studies sample from the same umbilical/placental vessels in case there are some as yet unidentified differences between these biofluids.

For a subset of samples, we performed measurement of FcRn in matched placenta. We found no significant correlation between placental levels of FcRn and the transfer rates of total IgG nor any of the antibody subclasses. One previous study has correlated placental FcRn with transfer ratios, and concluded that there was a positive association (37). However, this study measured “the percentage of the total area in the placental tissue with FcRn expression.” As the placental structure changes with gestation, including the proportions of the different cell types, measuring the percentage of tissue with FcRn content could be interpreted as just a measurement of syncytiotrophoblast surface area, rather than the concentration of FcRn within this tissue. Our measurement of FcRn by western blotting also has its limitations, given that it is measured in a mixed homogenate of placental tissue. Importantly however, we only looked at term placentas, which reduces the effect of gestational changes in placental structure. Our data suggests that inter-individual differences in transplacental transfer are mainly driven by differences in IgG, however this question warrants further investigation in a larger cohort, as well as investigation of other Fc receptors.

Finally, we explored the potential clinical variables that may impact on maternal and cord levels, and transfer ratios of total IgG and the IgG subclasses. Only a few variables impacted on the concentration of IgG in neonatal blood. Babies born at later gestation had significantly higher levels of IgG1 and IgG4. For IgG4 this appears to have been facilitated by significantly higher levels of IgG4 in the mothers, that was then passed to the fetus, rather than an increase in the rate of transplacental transfer (which was unchanged). For IgG1, maternal levels were no different with later gestation, and following adjustment for other associated variables, gestation was also not associated with the IgG1 transfer ratio. This suggests that the higher IgG1 at later gestation may reflect an accumulation of IgG1 in the fetal circulation over time, rather than an increased ability of the placenta to transfer IgG in the last weeks of term pregnancy. Several other studies have found a positive association between total IgG and antigen-specific IgG and gestation (11, 30–32). The main difference is that we studied healthy term (>37weeks) pregnancies, unlike other studies which looked at the effect of prematurity. Our study shows that even at term, every additional weeks' delay in delivery increases neonatal levels of IgG1 and IgG4.

Birth centiles were negatively correlated with neonatal levels of total IgG and of IgG2. This does not appear to be related to a change in maternal levels, nor lower transfer ratio of these subclasses, neither of which were associated with birth centiles. The reason for this is unclear, but it could be an effect of higher hemodilution of IgG in a larger fetal circulation. It would be difficult to explore this; perhaps an analysis that incorporated both infant birth centiles and placental weight could shed light on this observation. Unfortunately, we did not record placental size nor weight in this current study.

Babies born to rhesus negative mothers had significantly lower levels of IgG2. These mothers were given injections of anti-D IgG at 28 weeks gestation. There appeared to be no difference in maternal levels of IgG at birth, but there were significantly lower transfer ratios of IgG2 (but not of any other subclass). This suggests that the administered IgG could compete with transfer of endogenous IgG2, but a much larger cohort of rhesus negative pregnancies is required to study this further. There may be a benefit to ensuring a time interval between maternal vaccination and anti-D infusion, if IgG2 is important for protection, and maternal vaccination studies should include Rhesus status in their analyses.

Some maternal factors were associated with changes in maternal IgG levels and the maternal/cord transfer ratio, but had no effect on final IgG levels in the neonate. Non-white women had higher levels of IgG2 and IgG3, but the transfer ratios were lower in these pregnancies and thus cord levels were not significantly different. Older mothers had lower total IgG levels, but there was no effect on transfer nor cord levels. Interestingly, women who had previously had a live birth had higher levels of total IgG and IgG1 at birth. However, this was not reflected in neonatal blood, and the transfer ratio between maternal and cord blood was reduced in these pregnancies. Thus, as previously discussed, although maternal and cord IgG levels are positively correlated, the ability of the placenta to transfer IgG may be limited by potential saturation of Fc transport receptors at higher IgG concentrations. Previous studies found no effect of parity on the transfer of antigen-specific IgG in placental malaria nor HIV infection (36, 38), nor on cord blood antigen-specific antibody concentrations (39). The only other subclass transfer study relating to malaria and hypergammaglobulinemia also reported no effect of parity (17).

Finally, we investigated the impact of maternal vaccination on levels and transfer of IgG subclasses. Tdap vaccination status had no impact on maternal nor cord IgG concentrations. However, there was a positive correlation between later gestation at Tdap vaccination and higher transfer ratios of all four subclasses. This finding is of interest given that the recommended gestation for maternal Tdap/IPV vaccination has undergone several changes in the UK, partly as a result of studies showing that earlier vaccination results in higher anti-Tdap antibodies in infants. For example, a study by Eberhardt et al. (40) propose that the best cord blood levels of anti-Tdap antibodies occur when the vaccine is given between 26 and 29 weeks. However, despite this observation, the timing of Tdap/IPV was not associated with any significant changes in total/subclass IgG levels in cord blood, suggesting limited impact on bulk concentrations of IgG in the neonate. Perhaps more intriguing is our observation that babies born to influenza vaccinated mothers have significantly higher levels of IgG4. Vaccinated mothers also have higher IgG4 levels, which is interesting given that pregnancy has been previously associated with an elevated IgG4 response to seasonal influenza vaccine (41). We have previously shown that timing of influenza vaccination impacts on anti-influenza levels in babies, with the highest levels if mothers were vaccinated 4–24 weeks before delivery (42), but here we find no effect of influenza vaccine timing on neonatal levels nor transfer of the IgG subclasses.

In summary, the current study provides a detailed analysis and update on the transplacental transfer of IgG subclasses, including a review of 17 previous studies investigating maternal to fetal transfer of IgG subclasses. Most studies agree that IgG1 is the most efficiently transferred antibody subclass, with lower transfer of IgG2–4. We conclude that transfer of the IgG2–4 subclasses varies widely between study populations, but that IgG2 transfer may not be as poor as often stated. The diverse findings between studies could be due to differences in ethnicity, parity, vaccination status (including gestation of vaccination), gestation at delivery (even within term delivery populations) and birth centiles between study populations; clinical parameters that were associated with transfer ratios of total IgG and IgG subclasses from mother to fetus in our study. It is important that we continue to offer maternal vaccination to all women. Parous women, and those of non-white ethnicities may have reduced antibody transfer, and it may therefore be particularly important to engage with these demographic groups; we have previously reported maternal Tdap vaccination rates in our setting to be 36% lower in black women compared to white women (43). Encouragingly, neonatal levels of total IgG and IgG subclasses seems largely unaffected by maternal factors. However, administration of anti-D may result in lower IgG2 levels in the neonate, and maternal flu vaccination may result in higher levels of IgG4. Therefore, the impact of both of these interventions on the neonatal immune response should continue to be explored.

## Data Availability Statement

All datasets generated for this study are included in the article/Supplementary Material.

## Ethics Statement

The studies involving human participants were reviewed and approved by London-Hampstead and Yorkshire and the Humber Research Ethics Committees. Written informed consent to participate in this study was provided by all participants.

## Author Contributions

BD and SB contributed to patient recruitment and collection of clinical data. TC, TR, and MB processed the samples, carried out the laboratory investigations, and contributed to data preparation. GV performed the statistical analysis and contributed to figure and table preparation. BK and CJ contributed to study design and execution. BH conceived and designed the overall study and drafted the manuscript. All authors contributed to the drafting and revision of the manuscript.

## Conflict of Interest

The authors declare that the research was conducted in the absence of any commercial or financial relationships that could be construed as a potential conflict of interest.

## Supplementary Material

Supplemental Figure 1 Correlation between timing of maternal blood draw and maternal concentrations of total IgG and IgG subclasses profiles. Concentration of total IgG and IgG subclasses in women plotted against the timing of their blood draw. Women were bled 1 day before delivery (*n* = 7), on the day of delivery (*n* = 78), within ½ day of delivery (*n* = 2), within 1 day (*n* = 20), 2 days (*n* = 5) or 3 days (*n* = 3). Lines indicate simple linear regression (all non-significant at *p* > 0.05).

Supplemental Table 1 Descriptive statistics of maternal to fetal transfer of total IgG and IgG subclasses.
