# Supplementary figures and images for: Update on Transplacental Transfer of IgG Subclasses: Impact of Maternal and Fetal Factors
Source: Front Immunol. 2020 Sep 11;11:1920. doi: 10.3389/fimmu.2020.01920 (PMC7516031; doi:10.3389/fimmu.2020.01920)

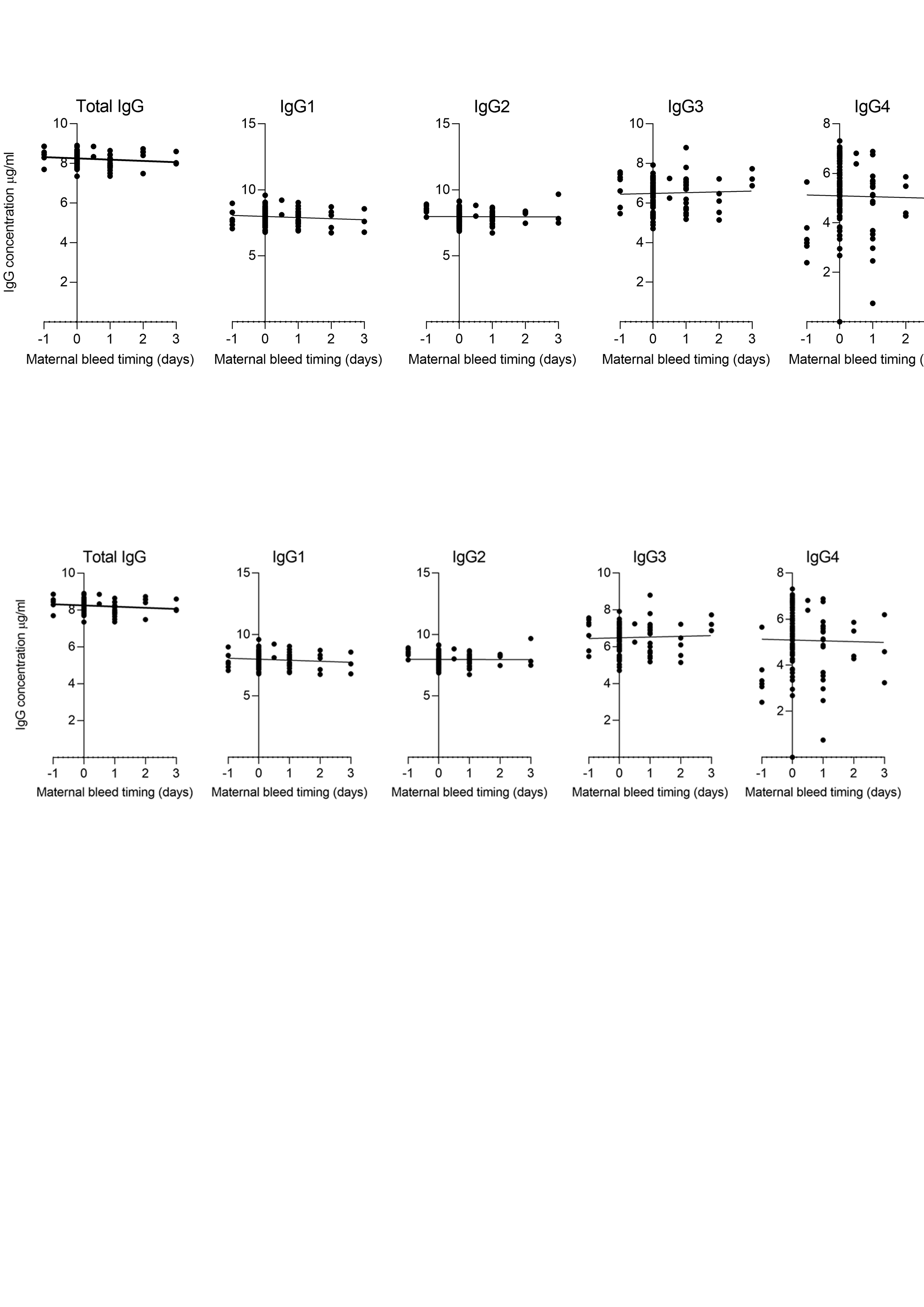

Supplement: Supplemental Figure 1 — Correlation between timing of maternal blood draw and maternal concentrations of total IgG and IgG subclasses profiles. Concentration of total IgG and IgG subclasses in women plotted against the timing of their blood draw. Women were bled 1 day before delivery (n = 7), on the day of delivery (n = 78), within ½ day of delivery (n = 2), within 1 day (n = 20), 2 days (n = 5) or 3 days (n = 3). Lines indicate simple linear regression (all non-significant at p > 0.05). [file Image_1.TIF]
