# Supplementary material for: Update on Transplacental Transfer of IgG Subclasses: Impact of Maternal and Fetal Factors
Source: Front Immunol. 2020 Sep 11;11:1920. doi: 10.3389/fimmu.2020.01920 (PMC7516031; doi:10.3389/fimmu.2020.01920)
Supplement: Supplemental Table 1 — Descriptive statistics of maternal to fetal transfer of total IgG and IgG subclasses. [file Table_1.docx]

**Supplemental Table 1.** Descriptive statistics of maternal to fetal transfer ratios for IgG subclasses 1-4

|  | IgG1 | IgG2 | IgG3 | IgG4 |
| --- | --- | --- | --- | --- |
| Mean | 1.538 ±0.6069 | 0.9326 ±0.4042 | 1.040 ±0.3148 | 0.9330 ±0.2566 |
| Median | 1.399 | 0.8511 | 0.9911 | 0.9334 |
| 25% percentile | 1.164 | 0.6859 | 0.8361 | 0.7897 |
| 75% percentile | 1.855 | 1.085 | 1.200 | 1.031 |
| Range | 0.3248 - 3.442 | 0.04719 - 3.071 | 0.2923 - 1.986 | 0.00219 - 1.798 |
